# Prognostic Value of miRNA-155 Expression in B-Cell Non-Hodgkin Lymphoma

**DOI:** 10.4274/tjh.2016.0286

**Published:** 2017-08-02

**Authors:** Ahmed M. L. Bedewy, Shereen M. Elmaghraby, Ahmed A. Shehata, Noha S. Kandil

**Affiliations:** 1 Alexandria University, Medical Research Institute, Alexandria Governorate, Egypt; 2 Alexandria University Faculty of Medicine, Department Internal Medicine, Alexandria Governorate, Egypt; 3 Alexandria University Faculty of Medicine, Department Chemical Pathology, Alexandria Governorate, Egypt

**Keywords:** MicroRNA-155, non-Hodgkin lymphoma, prognosis

## Abstract

**Objective:**

*MicroRNA-155 (miRNA-155)* resides within the B-cell integration cluster gene on chromosome 21. It can act either as an oncogene or as a tumor-suppressor gene, depending on the cell background in which *miRNA-155* is performing its specific target gene controlling function. Therefore, the aim of this study was to investigate *miRNA-155* expression in patients with B-cell non-Hodgkin lymphoma (NHL) and its relation to disease prognosis in diffuse large B-cell lymphoma (DLBCL) patients.

**Materials and Methods:**

Reverse transcription-polymerase chain reaction assay was performed to evaluate the expression levels of *miRNA-155* in 84 patients with newly diagnosed B-cell NHL and 15 normal controls.

**Results:**

Compared with normal controls, *miRNA-155* expression was significantly upregulated in patients. Moreover, higher levels of *miRNA-155* were associated with the presence of B symptoms, involvement of extranodal sites, and high Eastern Cooperative Oncology Group (ECOG) score. Higher levels of *miRNA-155* in DLBCL were associated with non-germinal B-cell-like type, the presence of B symptoms, involvement of extranodal sites, and higher International Prognostic Index (IPI) and ECOG scores. Only the high IPI score and high *miRNA-155* expression indicated a higher risk of lower event-free survival using multivariate Cox regression analysis. Our data demonstrated that the expression of *miRNA-155* was upregulated in newly diagnosed B-cell NHL patients. *miRNA-155* is expressed at a lower level in GCB-subtype DLBCL. Low IPI score and *miRNA-155* expression were predictors of longer event-free survival.

**Conclusion:**

Despite contradicting literature reports, the current findings suggest the potential value of *miRNA-155* as a biomarker of prognosis and monitoring in B-cell NHL, and especially that of the DLBCL type.

## Abstract (tr)

**Amaç:**

MikroRNA-155 (miRNA-155) 21. kromozom üzerinde B-hücresi birleşim kümesi içinde yer alır. miRNA-155 özgün hedef gen kontrolü yaptığı hücre zeminine göre onkogen veya tümor baskılayıcı gen olarak etkili olur. Bu nedenle, bu çalışmanın amacı B-hücreli Hodgkin dışı lenfoma hastalarında miRNA-155 ifadesini ve bunun yaygın büyük B hücreli lenfoma (YBBHL) olgularında hastalık prognozu ile ilişkisini araştırmaktır.

**Gereç ve Yöntemler:**

Seksen dört yeni tanı B-hücreli Hodgkin dışı lenfoma hastasında ve 15 normal kontrolde miRNA-155 ifade düzeyini değerlendirmek için ters transkripsiyon-polimeraz zincir reaksiyonu testi kullanıldı.

**Bulgular:**

Normal kontrol ile karşılaştırıldığında hastalarda miRNA-155 ifadesi belirgin artmıştı. Ayrıca, yüksek miRNA-155 düzeyleri B semptomlarının varlığı, nodal dışı bölge tutulumu ve yüksek Doğu Kooperatifi Onkoloji Grubu (ECOG) skoru ile ilişkiliydi. YBBHL olgularında yüksek miRNA-155 düzeyleri germinal-dışı B hücre fenotipi, B semptomları, nodal dışı bölge tutulumu, yüksek Uluslararası Prognostik İndeks (IPI) ve ECOG skorları ile ilişkiliydi. Çoklu değişkenli Cox regresyon analizinde sadece yüksek IPI skoru ve yüksek miRNA-155 ifadesi düşük olaysız sağkalım riski ile ilşkili bulundu. Bulgularımız yeni tanı YBBHL olgularında miRNA-155 ifadesinin arttığını gösterdi. YBBHL’nın germinal merkez alt tipinde miRNA-155 düşük düzeyde ifade oldu. Düşük IPI skoru ve miRNA-155 ifadesi uzun olaysız sağkalım için göstergeydi.

**Sonuç:**

Çelişkili literatüre verilerine karşın, mevcut bulgularımız miRNA-155’in prognostik bir biyobelirteç olarak başta YBBHL tipi olmak üzere B-hücreli Hodgkin dışı lenfomaların izleminde potansiyel değerine işaret etmektedir.

## INTRODUCTION

B-cell lymphomas constitute a heterogeneous group of lymphoproliferative neoplasms originating from B cells with a largely unknown pathogenesis. The current classifications of B-cell lymphomas are essentially based on the recognition of characteristic genetic abnormalities that deregulate the expression of oncogenes or tumor suppressor genes [1]. B-cell non-Hodgkin lymphomas (NHLs) are derived from mature B cells and account for approximately %70-90% of lymphoid neoplasms worldwide and 4% of all new cancers each year [2]. The most common types of NHL are diffuse large B-cell lymphoma (DLBCL) and follicular lymphoma, which together represent more than 60% of all cases [3,4].

MicroRNAs (miRNAs) are small, non-coding RNA stretches that consist of approximately 22 nucleotides. miRNAs function through post-transcriptional modulation of gene expression. This occurs by miRNA specifically binding to its target miRNA, thus inhibiting its translation into polypeptide [5]. The discovery of miRNA has exposed a new layer of gene expression regulation that affects many physiological and pathological processes of life [6]. Many abnormal miRNA expression patterns are found in various human malignancies, and certain miRNAs play roles as oncogenes or tumor suppressors [7]. The role of miRNAs in B-cell lineage development was reviewed by Fernando et al. [8]. Certain miRNAs have been found to characterize various subtypes of NHL and have important roles in B-cell differentiation and lymphomagenesis [9,10,11,12].

*miRNA-155* maps within the B-cell integration cluster gene on chromosome 21. It was suggested that *miRNA-155* can act either as an oncogene or as a tumor-suppressor gene, depending on the type of cell in which *miRNA-155* is performing its specific modulation of target gene expression [13]. However, no clinical correlation of *miRNA-155* and B-cell NHL was further investigated.

This work aims to investigate *miRNA-155* expression in patients with B-cell NHL and its relation to treatment response and disease prognosis in DLBCL patients.

## MATERIALS AND METHODS

Eighty-four patients with newly diagnosed histologically documented B-cell NHL, who presented to the Hematology Unit of the Internal Medicine Department of the Faculty of Medicine and the Hematology Department of the Medical Research Institute, Alexandria University, were included in the study. Informed consent was provided by all patients. The procedures followed were according to the ethical standards of the responsible committee on human experimentation (institutional and national) and with the Helsinki Declaration of 1975, as revised in 2008. Confidentiality of data was assured for all the patients. Fifteen subjects were enrolled in the study as healthy controls. History, clinical, and laboratory data of the studied B-cell NHL patients were collected, particularly age, sex, Eastern Cooperative Oncology Group (ECOG) performance status [14], presence of B symptoms, presence of bulky disease, involvement of extranodal sites, bone marrow infiltration, Ann Arbor clinical stage [15], serum lactate dehydrogenase (LDH) level, and the International Prognostic Index (IPI) score [16] in addition to treatment response and event-free survival for 54 DLBCL patients. DLBCL patients were treated with the standard CHOP regimen [17] and their response to treatment was assessed according to standard criteria [18]. The follow-up period of these patients ranged from 12 to 30 months with a median of 18.5 months.

Molecular study for the assay of *miRNA-155* in patients using quantitative real-time reverse transcriptase polymerase chain reaction (RT-PCR) was performed for both patients and healthy controls.

### RNA Extraction

Total RNA was isolated from 300 µL of cell-free serum using the mirVana™ miRNA Isolation Kit (Life Technologies, Carlsbad, CA, USA) according to the manufacturer’s instructions. RNA was dissolved in RNase-free water. The RNA concentration and purity were quantified with the NanoDrop ND-1000 (NanoDrop, Wilmington, DE, USA) and samples were stored at -80 °C until use.

### RT-PCR Quantification

Reverse transcription was performed using a First-Strand cDNA Synthesis Kit for miRNA (OriGene Technologies, Rockville, MD, USA) using 1 µg of extracted RNA according to the manufacturer’s instructions. Real-time PCR was performed using human *miRNA-155* and U22 qSTAR miRNA primer pairs and the SensiMix SYBR Master Mix Kit (OriGene Technologies) according to the manufacturer’s instructions using the StepOne real-time PCR system (Applied Biosystems, Foster City, CA, USA). Normalization was performed with U22 small nucleolar RNA expression. The 2^-ΔΔCt^ method was used in the analysis of PCR data. PCR efficiencies for *miRNA-155* and U22 were determined and were 98.1% and 97.8%, respectively [19].

### Statistical Analysis

Data were fed to a computer and analyzed using IBM SPSS 20.0. Comparisons between groups for categorical variables were assessed using the chi-square test. Multivariate logistic regression was assessed to find the factors most affecting event-free survival. A plotted event-free survival curve was used. Significance of the obtained results was judged at the 5% level.

## RESULTS

**Figure 1:**
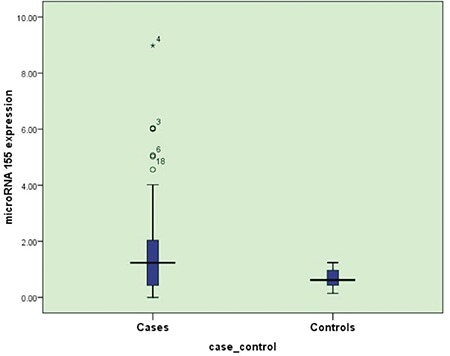
Boxplot graph of microRNA expression in patients and controls.

Compared to normal controls, *miRNA-155* expression was significantly upregulated in B-cell NHL patients (p=0.034) (Figure 1). miRNA expression in patients ranged from 0 to 8.98 relative expression units (REU) with a median value of 1.235 REU. NHL patients expressing *miRNA-155* at levels less than the median were assigned to the low-expression group (n=42), and those with expression equal to or above the median value were assigned to the high-expression group (n=42). High *miRNA-155* expression was associated with the presence of B symptoms, involvement of extranodal sites, and high ECOG performance score (Table 1). No association was found between *miRNA-155* expression and age, sex, or clinical stage.

Among the studied patients, 54 had DLBCL. The expression of *miRNA-155* in these DLBCL patients varied from high expression in 30 patients to low expression in 24 patients. Higher expression of *miRNA-155* was found in DLBCL patients who had the non-germinal B-cell type (31 cases) compared to the germinal center B-type (23 cases) (p=0.008). The presence of B symptoms, high IPI score, and high ECOG performance score were associated with higher *miRNA-155* expression (p=0.002, p=0.004, and p=0.006, respectively). The expression of *miRNA-155* was not associated with patients’ age (p=0.682), sex (p=0.902), serum LDH level (p=0.245), serum β2 microglobulin level (p=0.529), clinical stage (p=1.00), age-adjusted IPI score (p=0.338), extranodal involvement (p=0.088), or the response to treatment in DLBCL patients (p=0.800) (Table 2).

**Figure 2:**
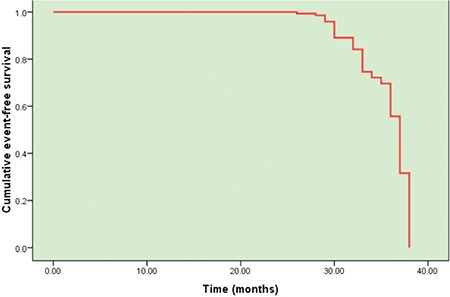
Event-free survival of the studied patients according to the studied covariates regression model.

**Figure 3:**
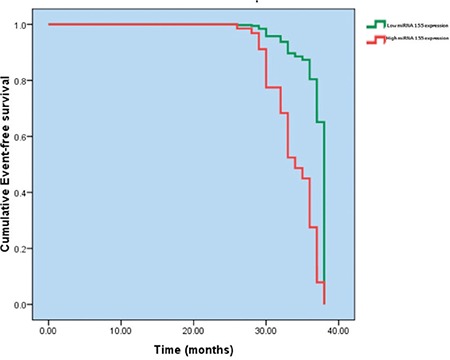
Event-free survival of the studied patients stratified by microRNA-155 expression (p=0.0035).

Multivariate Cox binary logistic regression analysis was performed to evaluate the influence of the studied factors on event-free survival among the studied DLBCL patients. Only high IPI score (odds ratio: 8.305) and high *miRNA-155* expression (odds ratio: 5.916) correlated with a higher risk of lower event-free survival (p=0.043 and p=0.035, respectively) (Table 3, Figures 2 and 3).

## DISCUSSION

Even though an explosion of molecular knowledge has paved the road for more precise recognition of distinct lymphoma subtypes, many patients still do not achieve satisfactory response to the available standard therapies [20]. Among many players, the instinctive molecular incongruity within each NHL type and the unclear discrimination of NHL types are important factors. As such, the identification of novel prognostic biomarkers and therapeutic targets can possibly offer a better outcome for each NHL patient. miRNAs represent an important category of putative biomarkers and therapeutic targets for NHL and other cancers.

*miRNA-155* overexpression was reported to contribute to tumorigenesis, possibly by dysregulating the expression of members of the PI3K-AKT pathway, the transforming growth factor beta (TGFβ) pathway, and other transcriptional regulators [21,22,23]. *miRNA-155* represses the SH2-domain encompassing inositol-5-phosphatase 1 (SHIP-1). SHIP-1 is a critical phosphatase that negatively down-modulates the AKT pathway. It exerts this action during normal B-cell development. Thus, sustained overexpression of *miRNA-155* in B cells is thought to unblock AKT activity, favoring B-cell proliferation. In concordance with this, Gironella et al. [24] ascribed this effect of *miRNA-155* to a blockade of caspase-3 activity and decreased tumor protein 53-induced nuclear protein 1 (TP53INP1), a nuclear protein capble of inducing cell cycle arrest and apoptosis through activation of caspase 3.

Interestingly, in DLBCLs, *miRNA-155* overabundance has been shown to induce resistance to the growth-inhibitory effects of both TGFβ1 and bone morphogenetic protein. This comes through the defective induction of p21 and the impaired cell cycle arrest caused by targeting SMAD5 [25,26]. This was supported by the work of Jiang and Aguiar [27] on DLBCL cell lines and a *miRNA-155* knock-out mouse model. They demonstrated that levels of the transcription factor SMAD5 are elevated in mature B lymphocytes, which exhibit an elevated sensitivity to TGFβ1 characterized by inhibition of retinoblastoma protein (RB) phosphorylation and a significant G0/G1 cell-cycle arrest.

In the present work, *miRNA-155* expression was significantly upregulated in patients with newly diagnosed B-cell NHL compared to controls (p=0.034). In concordance with this, Roehle et al. [28] reported that *miRNA-155* is overexpressed in follicular lymphoma and DLBCL when compared with normal lymph nodes. In addition, Shepshelovich et al. [29] and Thai et al. [30] found *miRNA-155* overexpression as a frequent finding in DLBCL patients.

In the present work, higher levels of *miRNA-155* in patients were associated with the presence of B symptoms, involvement of extranodal sites, and high ECOG performance score (p<0.001, 0.016, and 0.004, respectively). However, *miRNA-155* expression levels showed no association with sex, age, or clinical stage. In the DLBCL patients of the current series, higher levels of *miRNA-155* were associated with the presence of B symptoms, involvement of extranodal sites, high IPI score, high ECOG performance score, and non-germinal B-cell type. The expression of *miRNA-155* was not associated with sex, age, response to treatment, clinical stage, or serum markers (LDH and β2 microglobulin). Similar findings were reported by Malumbres et al. [10], Eis et al. [31], and Kluiver et al. [32], particularly regarding the association between *miRNA-155* expression and the non-germinal center B (GCB) immunophenotype of DLBCL. Using microarray analysis of prototypic cell lines, *miRNA-155* was more highly expressed in ABC-type than GCB-type cell lines and was overexpressed in de novo DLBCL (n=35), transformed DLBCL (n=14), and follicular center lymphoma cases (n=27) compared to normal B cells according to the report of Lawrie et al. [33]. On the other hand, Zhong et al. [34] reported a lack of association between *miRNA-155* expression levels and sex, age, clinical stage, or extranodal involvement.

The significant prognostic impact of *miRNA-155* expression together with the IPI score evidenced by Cox regression analysis in the present series was interestingly in line with the report of Zhong et al. [34]. They reported that low *miRNA-155* was associated with a longer 5-year progression-free survival in de novo DLBCL cases. They found that the expression levels of *miRNA-155* and IPI status were statistically significant independent indicators of prognosis (p<0.05) [28]. On the contrary, Lawrie et al. [33] reported the absence of an association between the expression of *miRNA-155* and prognosis (p=0.22). However, a recent report by Due et al. [35] confirmed the independent prognostic impact of *miRNA-155* in DLBCL and emphasized its potential value as a molecular tool in personalized medicine.

## CONCLUSION

Our data demonstrated that the expression of *miRNA-155* was upregulated in newly diagnosed B-cell NHL patients. *miRNA-155* is expressed at a higher level in ABC-type than in GCB-subtype DLBCL, suggesting that the quantiﬁcation of this miRNA may have a role in establishing the prognosis. Among the studied parameters, only low IPI score and low *miRNA-155* expression were predictors of longer event-free survival. Despite the contradicting literature reports in this regard, the current findings suggest the potential value of *miRNA-155* as a biomarker of prognosis and monitoring in B-cell NHL, especially for the DLBCL type.

## Figures and Tables

**Table 1:**
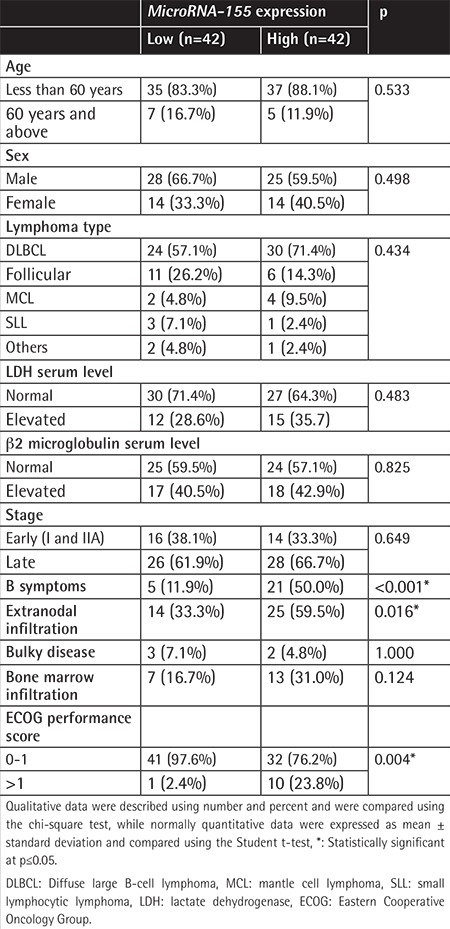
*MicroRNA-155* expression in patients as regards the studied parameters.

**Table 2:**
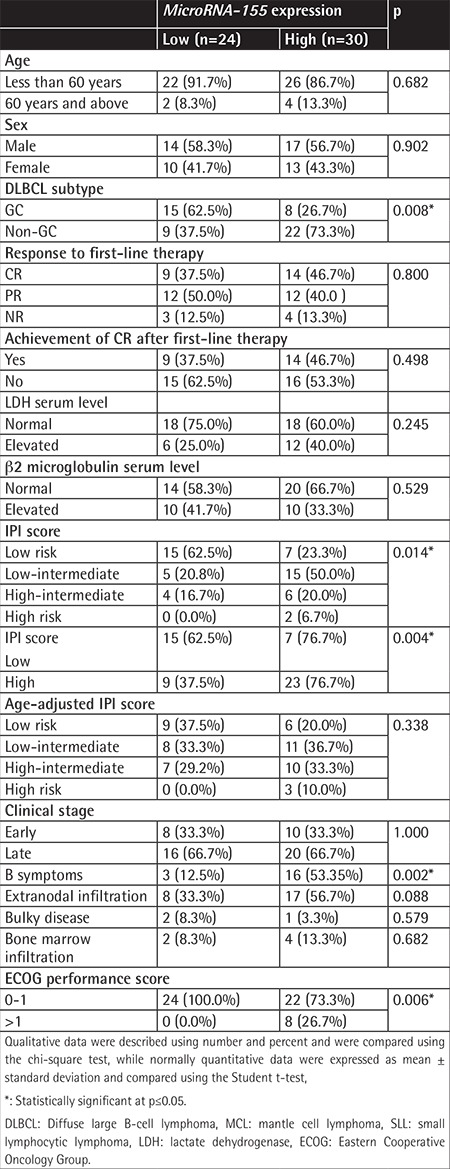
*MicroRNA-155* expression as regards the studied parameters in diffuse large B-cell lymphoma cases.

**Table 3:**
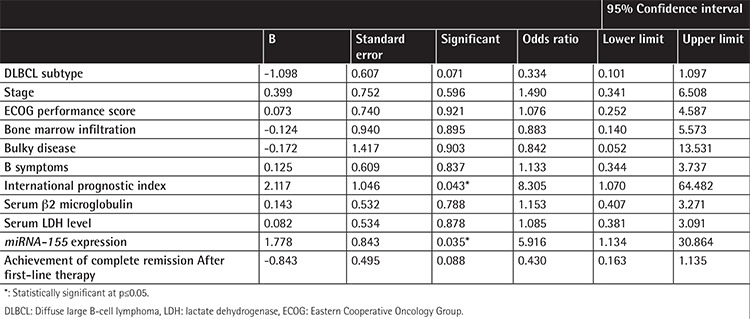
Multivariate binary logistic regression for the studied prognostic factors as regards event-free survival.
